# Supplementary material for: COUP-TFII regulates early bipotential gonad signaling and commitment to ovarian progenitors
Source: Cell Biosci. 2024 Jan 4;14:3. doi: 10.1186/s13578-023-01182-5 (PMC10768475; doi:10.1186/s13578-023-01182-5)
Supplement: Supplementary file 1 — Additional file 1: Figure S1. Expression pattern of NR2F2 and FOXL2projected on the UMAP plot showing cell lineages in the scRNA-seq datasets of female (A) and male (B) somatic cells obtained from human gonads between 6 and 21 weeks of gestation [24]. The color scale represents NR2F2 gene expression. CoelEpi, coelomic epithelium; OSE, ovarian surface epithelium; preGC, pre-granulosa cell; Gi, gonadal interstitial; Oi, ovarian interstitial; SMC, smooth muscle cell. Figure S2. (A) Transfected COV434 cells were blue fluorescence protein (BFP)-positive 24 h after transfection (excitation laser = 358 nm). (B) Fluorescence-activated cell sorting (FACS) was performed 48 h after transfection according to the filters P1: cell size and granularity settled for COV434 cell dimensions; P2: singlets; P3: enriched fluorescence intensity. Approximately 0.4% of the detected events corresponded to transfected single cells, which were seeded as single cells into wells of 96-well plates. (C) From day 17 after transfection, single cell-derived clones were observed under the phase contrast light microscope. Figure S3. (A) Principal component analysis (PCA) plot of WT (green dots) and NR2F2-KO COV434 (red triangles) replicates (n = 4/group) revealed an outlier sample in the WT group (arrow). (B) PCA plot considering n = 3 replicates for WT (outlier excluded) and n = 4 replicates for NR2F2-KO COV434 cells. Figure S4. (A) RT-qPCR validation of DEGs obtained by RNA-seq. RNA-seq fold-change (FC) and RT-qPCR FC of six genes when comparing the transcript expression between WT and NR2F2-KO COV434 cells. RNA-seq FC is based on the values of fragments per million mapped fragments (FPM). RT-qPCR FC represents the relative expression values (2−ΔΔCt) compared to the WT.S8 was used as a reference gene. (B) RT-qPCR results are shown as mean ± SEM (n = 2–4). Student’s t-test with Welch’s correction, *p < 0.05, **p < 0.01, ***p < 0.001, ****p < 0.0001. (C) UMAP of cell lineages in the scRNA-seq datasets [file 13578_2023_1182_MOESM1_ESM.docx]

Additional figures and tables

COUP-TFII regulates early bipotential gonad signaling and commitment to ovarian progenitors

Lucas G. A. Ferreira ^1,2^, Marina M. L. Kizys ^1^, Gabriel A. C. Gama ^1^, Svenja Pachernegg ^2,3^, Gorjana Robevska ^2^, Andrew H. Sinclair ^2,3^, Katie L. Ayers ^2,3^, Magnus R. Dias da Silva ^1,^*

^1^ Laboratory of Molecular and Translational Endocrinology (LEMT), Endocrinology Division, Department of Medicine, Escola Paulista de Medicina, Universidade Federal de São Paulo, São Paulo, Brazil

^2^ Murdoch Children’s Research Institute, Melbourne, Australia

^3^ Department of Paediatrics, The University of Melbourne, Melbourne, Australia.

*****Correspondence: [mrdsilva@unifes.br](mailto:mrdsilva@unifes.br)

**Table of Contents:**

Fig S1

Fig S2

Fig S3

Fig S4

Fig S5

Table S1

p.2

p.3

p.4

p.5

p.6

p.7


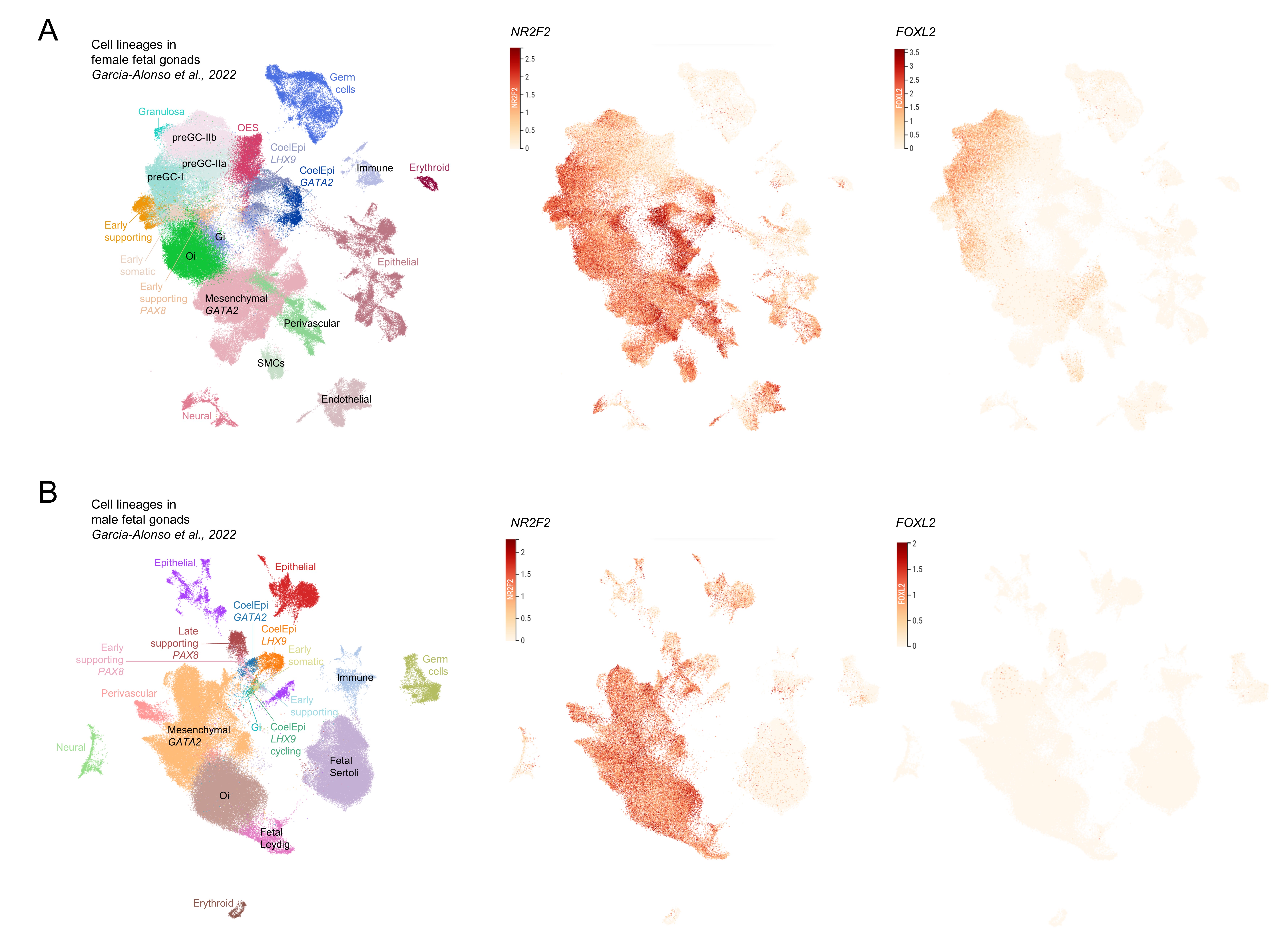


**Fig S1**. Expression pattern of *NR2F2* and *FOXL2* projected on the UMAP plot showing cell lineages in the scRNA-seq datasets of female (A) and male (B) somatic cells obtained from human gonads between 6 and 21 weeks of gestation (Garcia-Alonso et al., 2022). The color scale represents *NR2F2* gene expression. CoelEpi, coelomic epithelium; OSE, ovarian surface epithelium; preGC, pre-granulosa cell; Gi, gonadal interstitial; Oi, ovarian interstitial; SMC, smooth muscle cell.


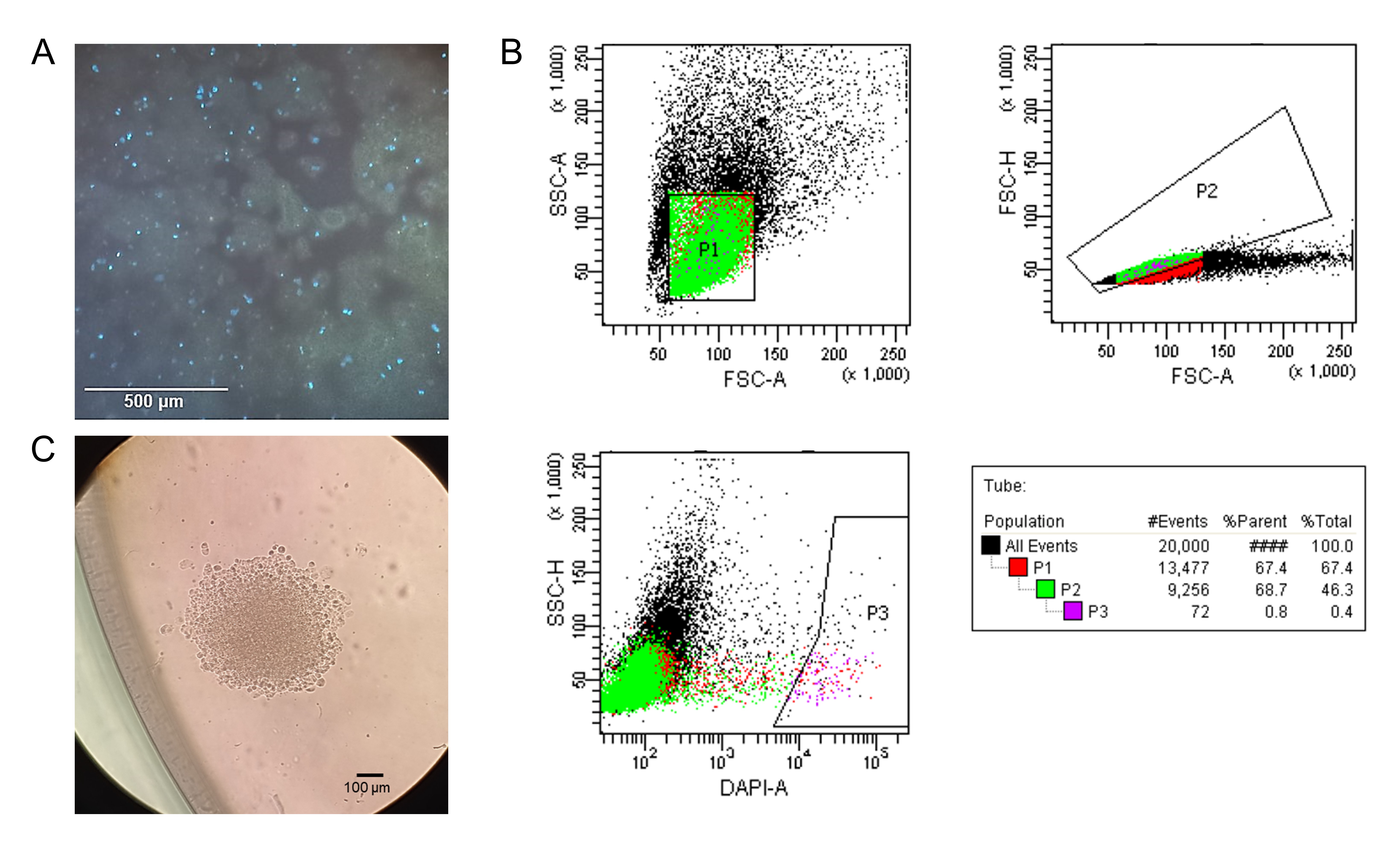


**Fig S2.** (**A**) Transfected COV434 cells were blue fluorescence protein (BFP)-positive 24 h after transfection (excitation laser = 358 nm). (**B**) Fluorescence-activated cell sorting (FACS) was performed 48 h after transfection according to the filters P1: cell size and granularity settled for COV434 cell dimensions; P2: singlets; P3: enriched fluorescence intensity. Approximately 0.4% of the detected events corresponded to transfected single cells, which were seeded as single cells into wells of 96-well plates. (**C**) From day 17 after transfection, single cell-derived clones were observed under the phase contrast light microscope.


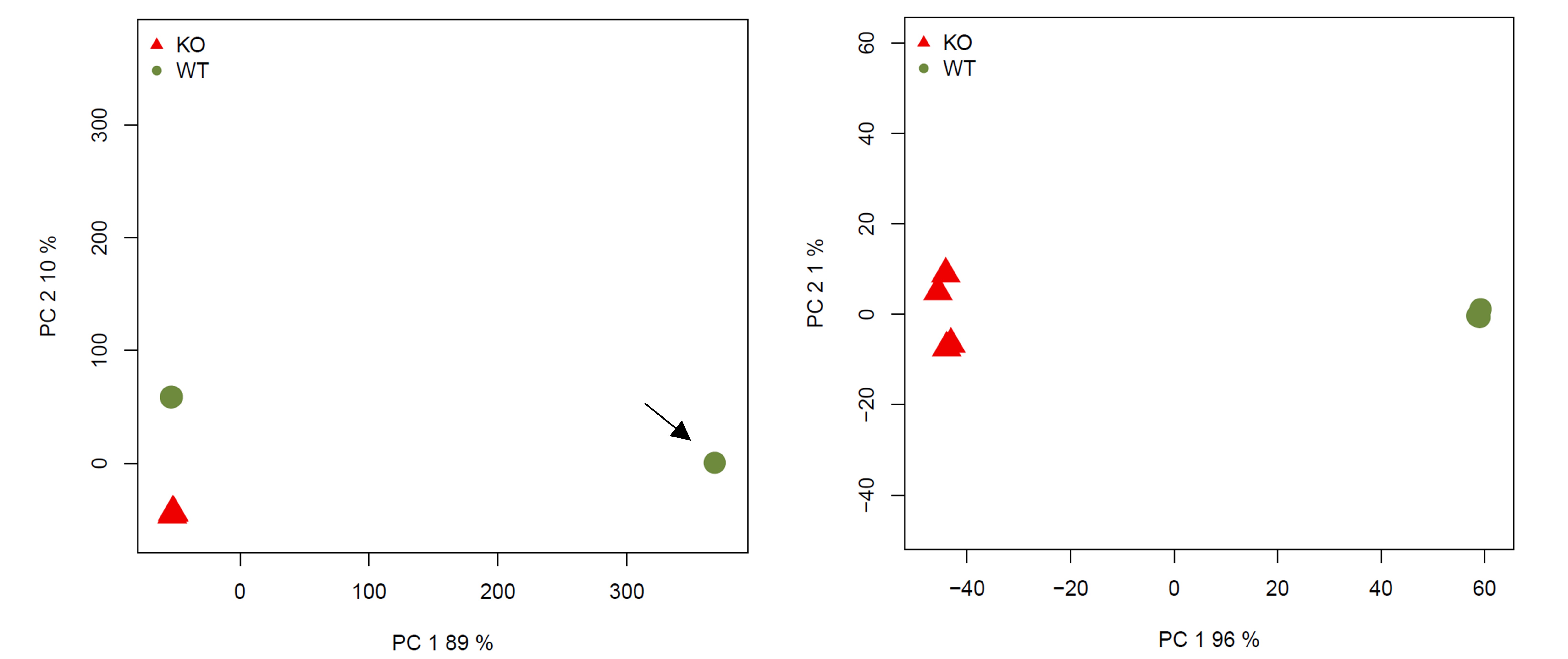


**Fig S3.** (**A**) Principal component analysis (PCA) plot of WT (green dots) and *NR2F2*-KO COV434 (red triangles) replicates (n=4/group) revealed an outlier sample in the WT group (arrow). (**B**) PCA plot considering n=3 replicates for WT (outlier excluded) and n=4 replicates for *NR2F2*-KO COV434 cells.


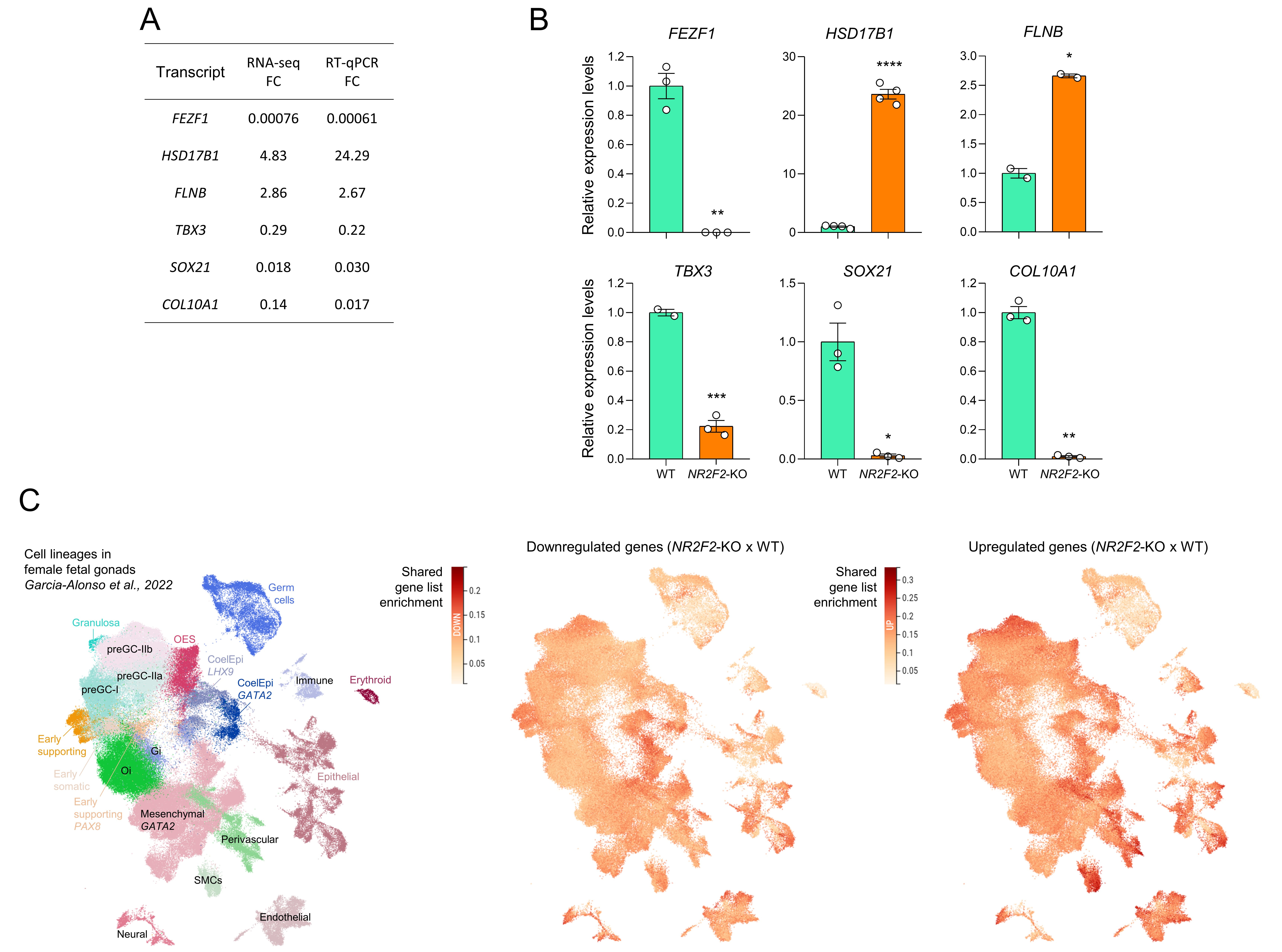


**Fig S4.** (**A**) RT-qPCR validation of DEGs obtained by RNA-seq. RNA-seq fold-change (FC) and RT-qPCR FC of six genes when comparing the transcript expression between WT and *NR2F2*-KO COV434 cells. RNA-seq FC is based on the values of fragments per million mapped fragments (FPM). RT-qPCR FC represents the relative expression values (2^−ΔΔCt^) compared to the WT. *S8* was used as a reference gene. (**B**) RT-qPCR results are shown as mean ± SEM (n=2-4). Student’s t-test with Welch's correction, *p<0.05, **p<0.01, ***p<0.001, ****p<0.0001. (**C**) UMAP of cell lineages in the scRNA-seq datasets of developing ovary and mesonephros obtained from human female fetuses between 6 and 21 weeks of gestation (Garcia-Alonso et al., 2022). The color scale represents the enrichment for downregulated and upregulated DEGs (*NR2F2*-KO *versus* WT COV434 cells). CoelEpi, coelomic epithelium; OSE, ovarian surface epithelium; preGC, pre-granulosa cell; Gi, gonadal interstitial; Oi, ovarian interstitial; SMC, smooth muscle cell.


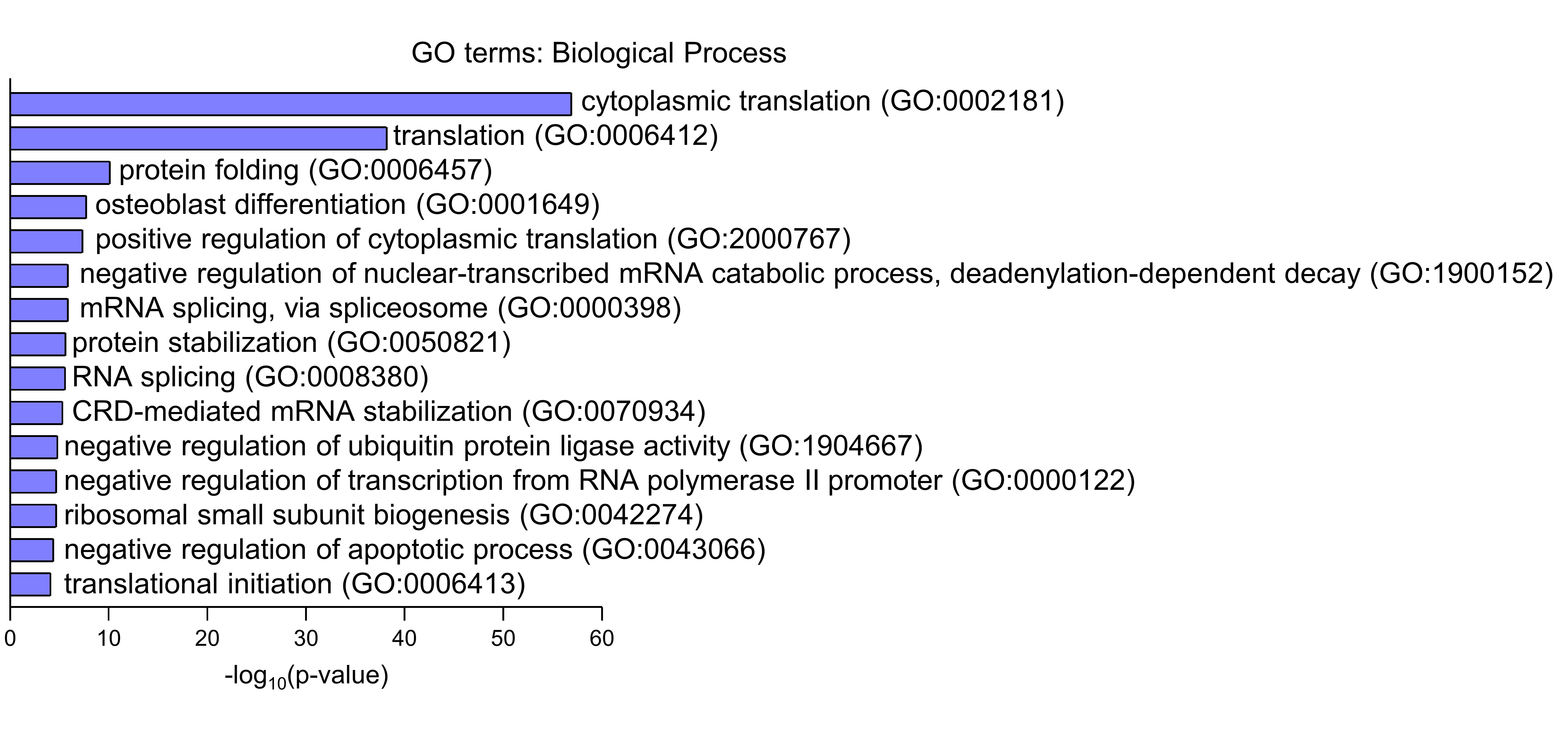


**Fig S5.** Gene ontology analysis of biological processes for the 378 overlapping genes between the 500 top expressed genes in WT and *NR2F2*-KO COV434 cells.

| **Gene/transcript target** | **Forward primer (5’-3’)** | **Reverse primer (5’-3’)** |
| --- | --- | --- |
| *Sanger sequencing* | | |
| *NR2F2* exon 2 | GAGGCTGGTCATTAACTGTGGA | AAGTTGCACTCAACAGTTTGGC |
| *RT-qPCR* | | |
| *COL10A1* | CAGGAACTCCCAGCACGCA | GGGTAGTGGGCCTTTTATGCC |
| *CTNNB1* | GTCGAGGACGGTCGGACT | AGGACTTGGGAGGTATCCACA |
| *E2F1* | CATCAGTACCTGGCCGAGAG | CCCGGGGATTTCACACCTTT |
| *FEZF1* | TCAGCCGAGGCTCTCCTAAT | TGCGCATTAAAGACCTTTCCAC |
| *FLNB* | GAAGGGAACAAAGAGGAGGCAC | GGAGATGTGCTGTCCTGCAA |
| *FOXL2* | TAAGCTCCTGTCGCTCCTCT | CTTTCCGCGGTGAATTTGGG |
| *FST* | CAGTTCATGGAGGACCGCAG | CCACAGTCCACGTTCTCACA |
| *HEY2* | AGATGCTTCAGGCAACAGGG | GCGCAACTTCTGTTAGGCAC |
| *HSD17B1* | TGGACGTGCTGGTGTGTAA | CGAACTTGCTGGCGCAATAA |
| *LHX9* | TGCCGAGCAGAAGACAACTC | CGAGGGCCAGCTTACATTCA |
| *NOTCH1* | ATATGCAGAACAACAGGGAGGA | TTGGCGGTCTCGTAGCTG |
| *NR2F2* v1 | GGTGTGCGGAGACAAGTCG | AGGTACGAGTGGCAGTTGAG |
| *NR2F2* v2 | TAGTGTGCAGGGTTTTCCAAGG |  |
| *NR2F2* v3 | GGGGTCCTGGGTACGTT |  |
| *NR2F2* v4 | GAGGCAAGGTGGCCAATTCTG |  |
| *PDGFB* | TGTCTCTCTGCTGCTACCTG | CCCATCTTCCTCTCCGGG |
| *S8* | AACAAGAAATACCGTGCCC | GTACGAACCAGCTCGTTATTAG |
| *SOX4* | GACCTGAACCCCAGCTCAAA | AGCCGGGCTCGAAGTTAAAA |
| *SOX9* | AAGAACAAGCCGCACGTCAA | CCGTTCTTCACCGACTTCCTC |
| *SOX21* | CGTTCATCGACGAGGCCAAG | ACCGGGAAGGCGAACTTGT |
| *TBX3* | GCATACCAGAATGATAAGATAACCC | TCAAACACCCTCATGGACTG |
| *WNT4* | CTCCACACTCGACTCCTTGC | CTGACCACTGGAAGCCCTGT |
| *Guide RNA for ablation of NR2F2 expression* | | |
| gRNA toward the exon 2 of *NR2F2* | CACCTGGTCAGCGCGAACTGCCCG | AAACCGGGCAGTTCGCGCTGACCA |

**Table S1. Primer and guide RNA sequences**
